# Fluid Biomarkers for the Antemortem Detection of Chronic Traumatic Encephalopathy: A Critical Review

**DOI:** 10.3390/neurolint18100195

**Published:** 2026-10-17

**Authors:** Nino Shatirishvili

**Affiliations:** Department of Chemistry and Biochemistry, San Diego State University, San Diego, CA 92182, USA; nshatirishvili@sdsu.edu

**Keywords:** chronic traumatic encephalopathy, traumatic encephalopathy syndrome, fluid biomarkers, repetitive head impacts, phosphorylated tau, neurofilament light chain, glial fibrillary acidic protein, S100B, sTREM2, antemortem diagnosis

## Abstract

Chronic traumatic encephalopathy (CTE) is a progressive tauopathy associated with repetitive head impacts (RHIs) and is currently confirmed only at autopsy. The absence of a validated antemortem diagnostic limits individual risk assessment, clinical trial enrollment, and differentiation from disorders such as Alzheimer’s disease (AD). This review critically evaluates five candidate fluid biomarkers: phosphorylated tau (p-tau), neurofilament light chain (NfL), glial fibrillary acidic protein (GFAP), S100 calcium-binding protein B (S100B), and soluble TREM2 (sTREM2). For each, the review assesses the biological mechanism, CTE/RHI-specific evidence, diagnostic performance, and major confounders. None currently provides CTE-specific discrimination. In the largest cohort, plasma p-tau217 tracked amyloid status rather than CTE pathology. NfL reflects axonal injury but is nonspecific and strongly age-dependent. GFAP is associated with clinical CTE certainty in football and combat-sport cohorts, although not in an independent rugby cohort. S100B responds acutely to head impacts without a persistent cumulative signal. sTREM2 may reflect microglial activation that is bidirectionally related to tau after RHI exposure, but its primary case–control comparison was null. Short study windows may also be poorly matched to CTE’s multi-decade latency, and most evidence for p-tau, NfL and GFAP derives from one cohort, with the total tau, extracellular-vesicle and sTREM2 findings from a second. The review therefore emphasizes multi-marker panels, assay standardization, and longitudinal antemortem-to-autopsy cohorts as the validation work required before any fluid biomarker can support antemortem CTE detection.

## 1. Introduction

Chronic traumatic encephalopathy (CTE) is a distinct neurodegenerative tauopathy caused by repetitive head impacts (RHIs), most commonly sustained through participation in contact sports or through military blast and combat exposure. The disorder was first documented nearly a century ago in boxers under the label “punch-drunk syndrome,” but it was not until the early 2000s, following high-profile neuropathological findings in deceased American football players, that CTE entered mainstream scientific and public consciousness. A 2009 clinicopathological series re-established CTE as a distinct progressive tauopathy following repetitive head injury [1], and subsequent work defined its pathological spectrum and a four-stage severity scheme [2]. A 2017 brain-bank series found CTE in 177 of 202 deceased American football players, including 110 of 111 former NFL players, while noting substantial ascertainment bias toward symptomatic donors [3]. Neuropathological diagnostic criteria have defined the pathognomonic lesion as perivascular accumulation of hyperphosphorylated tau (p-tau) in neurons, with or without astrocytes, at the depths of the cortical sulci, a distribution that distinguishes CTE from Alzheimer’s disease and other tauopathies [4].

Despite this diagnostic clarity at autopsy, CTE cannot currently be definitively diagnosed during life. This limitation defines the central problem this review addresses. Clinical research therefore relies on traumatic encephalopathy syndrome (TES), a research diagnostic construct formalized by NINDS in 2021. TES requires substantial RHI exposure, core clinical features of cognitive impairment and/or neurobehavioral dysregulation, a progressive course, and exclusion of conditions that better account for the clinical presentation [5]. TES criteria improve diagnostic consistency across research settings but remain a clinical inference rather than a biologically confirmed diagnosis, and recent validation work assessing TES against neuropathological ground truth across multiple brain banks has begun to quantify the sensitivity and specificity of this framework relative to autopsy-confirmed CTE stage and burden [6].

The scale of the underlying problem is substantial. A 2025 systematic review and meta-analysis pooling autopsy series of former contact-sport athletes found an overall CTE prevalence of 53.7% among individuals with a history of RHI exposure, with rugby athletes showing the highest subgroup prevalence at 64.7%, followed closely by American football athletes at 53.0% [7]. These figures, drawn exclusively from brain-bank cohorts enriched for individuals with known RHI exposure and possible selection bias toward symptomatic donors, likely overstate population-level prevalence, but they nonetheless underscore that RHI-related tauopathy is common enough among high-exposure populations to represent a public health concern rather than a rare curiosity. Compounding this, a growing body of evidence, including recent single-nucleus transcriptomic work demonstrating neuroinflammatory, vascular, and neuronal-loss signatures in RHI-exposed brain tissue independent of frank tau pathology, suggests that the pathophysiological cascade initiated by repetitive head trauma begins well before, and extends beyond, the tau deposition that defines the neuropathological diagnosis [8]. This raises the possibility that antemortem biomarkers indexing inflammation, glial activation, and axonal injury may hold diagnostic or prognostic value even in the absence of a validated in vivo tau signal.

The absence of a living diagnostic test has consequences extending beyond individual patient care. Without antemortem detection, clinicians cannot counsel athletes and military personnel on individualized risk, cannot titrate return-to-play or continued-service decisions to biological evidence of accumulating injury, cannot enroll biologically confirmed cases into therapeutic trials, and cannot disentangle CTE from the substantial diagnostic overlap it shares with Alzheimer’s disease, frontotemporal dementia, and normal aging in symptomatic older adults with a remote history of head trauma. The NIH-funded DIAGNOSE CTE Research Project, launched in 2015, was constructed explicitly to address this gap through multimodal biomarker development in a prospective cohort of former American football players [9], and its output, together with a growing body of independent literature, forms the evidentiary base this review draws upon.

Two broad antemortem biomarker modalities have been pursued in parallel: neuroimaging (structural and diffusion MRI, MR spectroscopy, susceptibility-weighted imaging, and tau- or inflammation-targeted PET) and fluid-based biomarkers assayed from blood or cerebrospinal fluid (CSF). Imaging modalities have been comprehensively reviewed elsewhere [10] and carry distinct methodological considerations concerning spatial resolution, radiotracer specificity, cost, and accessibility that merit separate treatment. Fluid biomarkers, by contrast, offer a scalable, minimally invasive, and comparatively low-cost avenue toward population-level screening, longitudinal monitoring, and trial enrichment, but the field remains fragmented across heterogeneous analytes, assay platforms, and study populations in a way that has not yet been synthesized into a single critical, cross-analyte account.

This article provides a critical review of five fluid biomarker analytes with substantial existing evidence bases in the CTE and RHI literature: phosphorylated tau (p-tau), neurofilament light chain (NfL), glial fibrillary acidic protein (GFAP), S100 calcium-binding protein B (S100B), and soluble TREM2 (sTREM2). The literature was identified through targeted searches of PubMed, PMC, and Google Scholar for the period January 2013 through July 2026, using combinations of the terms “chronic traumatic encephalopathy,” “traumatic encephalopathy syndrome,” and “repetitive head impact” with biomarker-specific terms. An analyte was included if it met all of the following criteria: (1) at least one peer-reviewed human study measured it in blood or CSF in relation to chronic or cumulative RHI exposure, or in a CTE-relevant clinical cohort, rather than solely in the acute post-injury window; (2) that study included a comparison group or a quantitative exposure or clinical outcome measure; and (3) it reported an effect size, a correlation coefficient, or a discrimination statistic such as an AUC. Because this is a narrative rather than a systematic review, these criteria define scope and do not constitute a PRISMA-style screening process. Other candidate analytes discussed elsewhere in the literature, including TDP-43, YKL-40, and UCH-L1, are represented in the literature mainly by acute TBI or concussion studies, or by CTE studies in brain tissue rather than biofluids, and at the time of writing no studies were identified meeting all three criteria in chronic RHI or CTE cohorts; this is a scope decision rather than a claim that these analytes lack future promise. Total tau (t-tau) is not treated as a separate analyte: the two plasma studies that meet these criteria [11,12] are reviewed alongside p-tau in Section 2.2, and CSF t-tau is discussed with sTREM2 in Section 6.2. For each of the five analytes reviewed, this review details the underlying biological mechanism, synthesizes the evidence linking the analyte to RHI exposure and/or clinical or neuropathological CTE outcomes, and critically evaluates specificity, assay-platform heterogeneity, and confounding factors that currently limit clinical translation. A comparative synthesis across analytes follows, together with the rationale and evidence for multi-marker panel approaches and an outline of the methodological and infrastructural steps—assay harmonization, longitudinal antemortem-to-autopsy cohorts, and ultrasensitive detection platforms—required to move fluid biomarkers from research tools to clinical assays.

Throughout, this review distinguishes four levels of evidence according to the reference standard against which a biomarker was assessed: (i) comparison of RHI-exposed and unexposed groups; (ii) association with TES diagnosis or TES-based CTE certainty; (iii) association with comorbid pathology such as amyloid-PET status; and (iv) correlation with neuropathologically confirmed CTE at autopsy. TES remains a clinical research construct whose correspondence with postmortem diagnosis is only moderate [6], so findings at levels (i)–(iii) should not be read as evidence of CTE detection. As summarized in the comparative synthesis (Section 7), almost all current fluid biomarker evidence falls within levels (i)–(iii); level (iv) data are limited to small postmortem subsamples [13]. The reference standard for each finding is stated explicitly throughout the analyte sections that follow.

## 2. Phosphorylated Tau (p-tau)

### 2.1. p-tau: Rationale and Biological Mechanism

Because hyperphosphorylated tau is the pathognomonic substrate of CTE at autopsy, fluid-phase p-tau was the earliest and most intuitively motivated candidate for an antemortem proxy. Multiple phosphorylation-site-specific epitopes have been developed as plasma and CSF assays, most prominently p-tau181, p-tau217, and p-tau231, each reflecting distinct kinase activity and conformational states of the tau protein and each validated to varying degrees against amyloid-beta (Aβ) and neurodegeneration pathology in the Alzheimer’s disease (AD) literature before being applied to RHI-exposed cohorts. This borrowed validation history is itself a methodological complication that recurs throughout this section: essentially all p-tau assays in current use were developed and calibrated to detect AD-type co-pathology, not the distinct sulcal, perivascular p-tau conformation characteristic of CTE, and the degree to which these assays cross-react with or are blind to CTE-specific tau remains incompletely resolved.

### 2.2. p-tau: Evidence in RHI and CTE Cohorts

The most methodologically robust data come from the DIAGNOSE CTE Research Project, a multicenter, NIH-funded prospective cohort comparing former professional and collegiate American football players with asymptomatic, unexposed controls [9]. Preliminary findings from this cohort were first reported in conference-abstract form [14] and subsequently published as a full peer-reviewed case–control study [13]; the two references describe the same underlying analysis and are cited together here. In the final published analysis of plasma p-tau217, 177 former football players and 54 unexposed participants were included. Former players showed modestly higher baseline concentrations, but this difference was driven in part by a higher proportion of amyloid-PET-positive participants, indicating an important contribution from comorbid AD-type pathology rather than an RHI-specific tau signal. Plasma p-tau217 performed comparably to established CSF Aβ-pathology markers for identifying amyloid-PET positivity (p-tau217 AUC = 0.88, 95% CI 0.80–0.96; CSF p-tau181/Aβ42 AUC = 0.89, 95% CI 0.79–1.00; CSF Aβ40/42 AUC = 0.85, 95% CI 0.72–0.98), but this strong performance was specific to detecting Aβ pathology, not CTE itself. In the postmortem subsample (*n* = 9 brain donors, 6 with neuropathologically confirmed CTE stages II–IV and none with co-occurring Alzheimer’s disease), seven donors had negative or intermediate p-tau217 concentrations that were concordant with their Aβ-PET status. Two donors with stage III CTE had elevated p-tau217 values on the high-molecular-weight assay but normal concentrations on additional testing with a brain-derived low-molecular-weight assay, indicating that these apparent outliers were assay-dependent. Overall, antemortem p-tau217 did not correspond to CTE diagnosis or stage. Separately, p-tau217/Aβ-PET discordances occurred in the broader cohort, underscoring that these are distinct comparisons. The study concluded that plasma p-tau217 is unlikely to be useful for CTE detection, though it may help identify or rule out comorbid Aβ pathology in this population. In a longitudinal, repeated-measures analysis testing a time-by-group interaction, rather than a single cross-sectional comparison, p-tau217 additionally showed no significant association with TES diagnosis, TES-based CTE certainty level, or quantitative RHI exposure metrics within the football-player subgroup [13,14].

A separate analysis from the same broader research program (180 former football players vs. 60 asymptomatic, unexposed male participants aged 45–74), examining plasma p-tau181 and p-tau231 alongside other analytes in former elite American football players, found significantly elevated p-tau181 and p-tau231 relative to unexposed controls (padj = 0.016), with a corresponding reduction in the Aβ42/p-tau181 ratio (padj = 0.004). However, discrimination accuracy was only modest (area under the receiver operating characteristic curve [AUC] = 0.742; acceptable group-level discrimination, insufficient for individual diagnosis), and the authors explicitly noted that available p-tau epitopes at the time of that study were not optimized to detect RHI-specific pathology, recommending further evaluation of newer-generation epitopes such as p-tau217 [15]. Older, smaller studies examining CSF- and plasma-derived extracellular vesicle (EV) fractions in former NFL players (*n* = 21 former players vs. age-matched controls for the plasma EV study) have reported similarly modest discrimination (AUC 0.72–0.74 for EV-derived total tau and p-tau181), though a combinatorial panel of three non-tau EV proteins (collagen VI alpha-3 and alpha-1 chains and reelin) achieved substantially higher discrimination (AUC = 0.85, 85% accuracy) in the same cohort, an early proof of concept for the multi-marker panel approach discussed further in Section 8 [16,17].

Two earlier studies from the Diagnosing and Evaluating Traumatic Encephalopathy using Clinical Tests (DETECT) cohort of former NFL players, the same cohort that supplied the EV [16,17] and sTREM2 [18] data, measured tau outside the phospho-epitope framework. In 96 former NFL players and 25 controls, plasma t-tau did not differ between groups; within the former players, a higher cumulative head impact index predicted higher plasma t-tau (*p* = 0.0137), and concentrations of 3.56 pg/mL or above were observed only in former players, but t-tau did not predict clinical function [11]. In 78 former NFL players and 16 controls, plasma exosomal tau was higher in former players (*p* < 0.0001), separated the groups with 82% sensitivity and 100% specificity, and correlated with poorer memory and psychomotor speed [12]. The control groups were small, the exosomal tau study was explicitly preliminary, and neither finding has been replicated outside DETECT or compared with neuropathology.

Evidence independent of both American football cohorts comes from rugby. In 200 former elite rugby players (median age 44 years) and 33 unexposed controls from the Advanced Brain Health Clinic cohort, plasma p-tau217 was 17.6% higher in former players (95% CI 3.7–33.3) and exceeded the control 97.5th centile in 23.1% of them; 24 former players (12%) met TES criteria, none had dementia, and elevated p-tau217 was associated with 2.8-fold higher odds of TES (95% CI 1.1–7.1) [19]. A companion multiplex proteomic analysis of the same cohort reported higher p-tau181 in former forwards [20]. These findings sit at evidence levels (i) and (ii): the control group was small, participants were self-referred, and no autopsy data are available.

### 2.3. p-tau: Critical Appraisal

Three limitations recur across the literature and warrant explicit emphasis. First, AD co-pathology is a major interpretive confound for p-tau biomarkers, particularly p-tau217, although not all reported p-tau181/p-tau231 differences in RHI cohorts have been explained by AD-related pathology [13,15]. Any p-tau elevation detected in an RHI-exposed cohort should therefore be interpreted alongside Aβ-PET or CSF Aβ42/40 status before it is attributed to CTE specifically. Indeed, the strongest reported discrimination for p-tau217 in the literature (AUC 0.88) concerns detection of Aβ pathology rather than CTE. Second, the direct tau measures studied to date show only modest discrimination between RHI-exposed and unexposed groups (AUC 0.742 for p-tau181/p-tau231 [15]; AUC 0.72–0.74 for EV-derived tau species [16,17]) and have not demonstrated validated diagnostic performance for CTE. The higher-performing 0.85-AUC EV panel reported in earlier work consisted of non-tau proteins and should therefore be interpreted as evidence for combinatorial approaches rather than as evidence of p-tau performance [16,17]. Third, and most fundamentally, no routinely available fluid p-tau assay has demonstrated selectivity for the CTE-specific tau fold identified at autopsy as opposed to tau phosphorylation patterns shared across tauopathies. Until assays with demonstrated selectivity for CTE-relevant tau species are developed and validated, plasma and CSF p-tau are likely to remain more useful for identifying comorbid AD pathology than for establishing CTE itself. The rugby finding that elevated p-tau217 accompanies higher odds of TES [19] qualifies this conclusion without overturning it: it rests on a clinical syndrome rather than neuropathology, has not been replicated, and contrasts with the absence of a p-tau217-TES association in DIAGNOSE CTE [13].

### 2.4. Toward Conformation-Selective Tau Detection

Structural work has established that CTE tau is molecularly distinct from AD tau. Cryo-electron microscopy of tau filaments from three individuals with CTE revealed a fold that was identical across cases but differed from those of Alzheimer’s and Pick disease filaments, including a distinct beta-helix conformation enclosing a hydrophobic cavity occupied by a non-tau density, possibly a cofactor [21]. This provides a structural rationale for assays that recognize CTE-specific conformers rather than phosphorylation sites shared across tauopathies.

Seed amplification assays are currently the most advanced route toward conformation-sensitive detection. Tau real-time quaking-induced conversion (RT-QuIC) exploits the ability of pathological tau aggregates to template the fibrillization of recombinant tau substrates, detecting femtogram quantities of seeds [22]. However, existing 3R/4R assays detect CTE seeding activity in brain homogenate without clearly separating CTE from AD, because both diseases aggregate mixed 3R/4R tau isoforms [22,23]. In one assay, CTE brain seed concentrations were comparable to those of the weakest AD specimens [22]. Discrimination of Pick disease from AD and CTE has been achieved, but discrimination of CTE from AD has not [23]. To the author’s knowledge, no study has yet demonstrated CTE-selective tau seeding activity in antemortem CSF or blood.

Three developments would move this approach toward clinical use: substrates or readouts that resolve CTE from AD conformers; validation in antemortem biofluids from cohorts with subsequent autopsy confirmation; and testing in individuals with mixed CTE and AD pathology, which is common in older RHI-exposed populations. Until these are achieved, conformation-selective tau detection remains a research objective rather than an available biomarker, and this gap underlies the specificity limitation affecting every p-tau finding reviewed above (Section 8.4).

## 3. Neurofilament Light Chain (NfL)

### 3.1. NfL: Rationale and Biological Mechanism

Neurofilament light chain is a structural cytoskeletal protein released into extracellular fluid upon axonal injury, and it is among the best-validated fluid biomarkers of neuroaxonal damage across the neurodegenerative disease spectrum, with established diagnostic and prognostic value in multiple sclerosis, amyotrophic lateral sclerosis, and Alzheimer’s disease. Unlike p-tau, NfL carries no inherent proteinopathy specificity: it rises in response to axonal injury of essentially any cause, including normal aging itself, which positions it as a sensitive, but fundamentally nonspecific index of neurodegenerative burden rather than a CTE-specific marker. Its candidacy in the CTE literature rests less on biological specificity and more on the hypothesis that cumulative RHI exposure produces measurable diffuse axonal injury independent of, or in addition to, focal tau deposition, a hypothesis consistent with the diffusion-imaging literature showing white matter microstructural disruption in RHI-exposed cohorts.

### 3.2. NfL: Evidence in RHI and CTE Cohorts

Within the DIAGNOSE CTE cohort, plasma NfL was examined as a secondary marker of neurodegeneration in an analysis primarily focused on neuroinflammatory correlates of neurobehavioral dysregulation (NBD), one of the two core clinical features of TES [24]. Plasma and CSF NfL correlated moderately well with one another, supporting plasma as an adequate proxy for the less accessible CSF compartment. Critically, plasma NfL was associated with several NBD symptom domains (emotional dyscontrol, impulsivity, and total NBD burden), but only within the older-age subgroup (≥60 years) of former players; no such association was detected in the cohort as a whole or in younger players. This is an age dependence consistent with NfL’s well-established tendency to rise with normal aging and raising the question of whether the older-group signal reflects RHI-specific pathology, age-related neurodegeneration more broadly, or an interaction between the two that current cross-sectional designs cannot disentangle [24]. Plasma NfL in this same analysis was also inversely associated with executive function and processing speed, suggesting it tracks a broader dysexecutive-behavioral syndrome rather than a narrow, RHI-specific signature [24].

A separate plasma biomarker panel study in former elite American football players found that younger age of first exposure (AFE) to football was associated with higher plasma NfL, an association that, notably, was itself only detectable in older participants at the time of sampling, again pointing to an interaction between cumulative exposure, aging, and current NfL concentration rather than a simple linear dose–response relationship. In the same study, quantitative RHI exposure proxies (career length, cumulative head impact index) were not consistently associated with NfL concentrations after correction for multiple comparisons, a null finding that recurs across several DIAGNOSE CTE analyses and tempers optimism that NfL directly indexes cumulative mechanical exposure in a way that could be used for individual-level exposure quantification [15].

### 3.3. NfL: Critical Appraisal

NfL’s principal strength, its well-characterized, cross-validated relationship to axonal injury in general, is simultaneously its principal weakness as a candidate CTE biomarker: because it is elevated in essentially any neurodegenerative or acute neurological insult, an elevated NfL value in an RHI-exposed individual cannot on its own distinguish CTE-related axonal injury from age-related decline, comorbid cerebrovascular disease, or an unrelated neurological process, and the pronounced age-dependence observed across DIAGNOSE CTE analyses means that age-stratified or age-adjusted reference ranges are not merely advisable, but essential to any future clinical application. The consistent failure of quantitative RHI exposure metrics to correlate robustly with NfL concentration is also a meaningful negative finding: if NfL fails to track a dose–response relationship with mechanical exposure, its utility may lie less in exposure quantification and more as a nonspecific adjunct within a multi-marker panel, flagging general neurodegenerative burden that must be interpreted alongside more disease-specific analytes. NfL is best characterized, at this stage of the evidence, as a sensitive, but non-diagnostic marker: useful for detecting that neurodegeneration is occurring, but silent on whether CTE is its cause.

## 4. Glial Fibrillary Acidic Protein (GFAP)

### 4.1. GFAP: Rationale and Biological Mechanism

GFAP is the principal intermediate filament protein of the astrocyte cytoskeleton and is released into extracellular fluid and subsequently the bloodstream upon astrocytic injury and reactive astrogliosis. It is a brain-enriched protein with minimal physiological presence in peripheral biofluids, a property that supports its utility as an index of CNS astrocytic activation even though it carries no proteinopathy specificity of its own. GFAP has an established clinical track record in acute TBI, where it rises rapidly after injury; in combination with UCH-L1, GFAP is incorporated into blood-based tests authorized by the FDA through the De Novo pathway; these tests are used with other clinical information to aid evaluation and CT triage in adults with suspected mild TBI [25]. It is also validated as a marker of astrogliosis and disease progression in Alzheimer’s disease, Parkinson’s disease, and multiple sclerosis. Its candidacy in CTE rests on a biologically plausible mechanism: astrocytic tau deposition and reactive astrogliosis are recognized, if secondary, components of CTE neuropathology alongside the primary neuronal perivascular p-tau lesion, giving GFAP a somewhat more direct pathological rationale in CTE than its role in acute TBI alone would suggest.

### 4.2. GFAP: Evidence in RHI and CTE Cohorts

A prominent CTE-relevant finding comes from the plasma biomarker analysis of former elite American football players discussed in Section 2 and Section 3: plasma GFAP was significantly higher in participants classified as TES-CTE possible/probable than in those classified as TES-CTE no/suggestive [15]. This represents a difference between grouped clinical-certainty categories, not evidence of a monotonic graded increase across every certainty level. The same study found that younger age of first exposure to football was associated with higher plasma GFAP, with the association concentrated in older participants at the time of sampling, suggesting a possible age-exposure interaction rather than a simple linear dose–response relationship [15]. Importantly, evidence now extends beyond former football players. In a study of 192 professional combat athletes from the Professional Athletes Brain Health Study, progressively higher GFAP concentrations were associated with greater odds of a TES diagnosis, whereas NfL and p-tau231 were not significantly related to TES [26]. This broadens the clinical evidence for GFAP, although TES remains an imperfect surrogate for neuropathologically confirmed CTE.

Findings elsewhere in the literature complicate this otherwise promising picture. A study of active-season German American football players found that a single season of repetitive subconcussive and concussive impacts did not produce measurable pre- to post-season changes in serum GFAP, p-tau, NfL, or S100B relative to non-playing controls [27]. As discussed in Section 5.2, no null result of this kind establishes a defined accumulation threshold; rather, it highlights the importance of sampling timing, biomarker kinetics, cohort characteristics, and the distinction between acute responses and long-latency neurodegenerative outcomes. A separate CSF biomarker analysis from the DIAGNOSE CTE cohort (180 male former football players: 120 professional, 60 college) examining supportive psychiatric features of TES (depression, anxiety, apathy) found that these nonspecific psychiatric symptoms correlated variably with generic markers of neurodegeneration and microglial activation; that analysis did not examine GFAP specifically and is discussed here only for what it shows about TES as a benchmark [28]. This underscores a central limitation of current validation work: biomarkers are often benchmarked against TES, a clinical research syndrome that remains an imperfect proxy for neuropathological ground truth.

Independent rugby data do not reproduce the GFAP signal. In the Advanced Brain Health Clinic cohort, plasma GFAP did not differ significantly between former elite rugby players and controls in the primary biomarker analysis [19] and was lower in former players (fold change 0.77) in the companion multiplex proteomic analysis, in which biomarker profiles were unrelated to TES classification [20]. The authors regarded the basis of the lower GFAP as uncertain. These participants were in mid-life (median age 44 years), and the discrepancy may reflect age, sport or assay platform; nonetheless, it shows that the GFAP association has not yet generalized across RHI populations.

### 4.3. GFAP: Critical Appraisal

GFAP currently has one of the more consistent clinical associations among the fluid analytes reviewed here, with supportive findings in both former football players and professional combat athletes [15,26]. This consistency does not extend to rugby, where GFAP was not raised in former players and was lower than in controls on a multiplex platform [19,20]. Nevertheless, neither study validates GFAP against neuropathologically confirmed CTE, and GFAP remains nonspecific to CTE relative to Alzheimer’s disease, other neurodegenerative disorders, and general astroglial injury. The null finding from the single-season German cohort further illustrates that acute-phase GFAP kinetics after individual TBI events do not straightforwardly translate into a chronic RHI signal [27]. More recently, a 2026 analysis integrating cognitive performance, blood biomarkers (including GFAP, NfL, total tau, and p-tau231), APOE-ε4 status, and structural MRI across two independent RHI-exposed cohorts achieved multidomain AUCs of 0.91 in professional fighters and 0.84 in former football players for TES classification [29]. This is consistent with integrated biomarker frameworks, subject to the incorporation bias discussed in Section 8.2, but does not establish standalone GFAP discrimination or an antemortem diagnostic for CTE. Independent validation against autopsy-confirmed disease therefore remains essential.

## 5. S100 Calcium-Binding Protein B (S100B)

### 5.1. S100B: Rationale and Biological Mechanism

S100B is a calcium-binding protein produced predominantly by astrocytes and, under normal physiological conditions, largely confined to the CNS, with cerebrospinal fluid concentrations roughly 100-fold higher than serum. Its appearance in peripheral blood is classically understood as a marker of blood–brain barrier (BBB) disruption, since S100B does not appreciably cross an intact barrier; its detection in serum after head impact has therefore been interpreted as direct evidence of transient BBB compromise rather than a signal specific to any particular downstream neuropathological process [30]. S100B carries the longest and most clinically mature track record of any analyte reviewed in this article: it has been extensively studied in acute mild TBI, is incorporated into some emergency-department clinical decision guidelines to help rule out the need for head CT after minor head injury, and is among the biomarkers described as approaching clinical validation for concussion assessment more broadly [30]. This maturity, however, was established almost entirely in the acute, single-injury TBI context, and its extension to chronic, cumulative RHI exposure and CTE rests on a considerably thinner and more indirect evidence base [31].

### 5.2. S100B: Evidence in RHI and CTE Cohorts

The most substantive line of RHI-specific S100B evidence comes not from CTE case–control comparisons, but from studies of repeated subconcussive BBB disruption in active football players who had not sustained a diagnosed concussion. In one such study, serial pre- and post-game serum sampling across a season in 67 players from three college football teams demonstrated repeated S100B surges consistent with recurrent, subclinical BBB disruption, and a subset of players who developed serum autoantibodies against S100B showed persistent diffusion tensor imaging (DTI) white matter abnormalities that correlated with cognitive changes [32]. This autoantibody-mediated pathway is mechanistically distinct from the p-tau, NfL, or GFAP processes reviewed above and represents a candidate mechanism linking repetitive subconcussive impact to downstream white matter injury, although it has not been directly linked to CTE neuropathology at autopsy [32,33]. A separate prospective study enrolled 17 high school football players and analyzed longitudinal biomarker data from 15 players. Serum S100B increased acutely after games without diagnosed concussion, and the magnitude of the pre- to post-game increase was associated with head-impact frequency and kinematics. However, pre- and post-season S100B concentrations did not differ, indicating that S100B did not detect cumulative season-long burden in that study [34].

Consistent with this distinction between acute and cumulative signals, the single-season German American football cohort discussed in Section 4 found no significant pre- to post-season change in serum S100B [27]. Taken together with the high school study, these findings suggest that S100B is more clearly supported as an acute, temporally proximal marker of head-impact-associated BBB or astroglial perturbation than as a persistent marker of cumulative RHI burden. Therefore, failure to detect a pre- to post-season change should not be interpreted as proof of a long biological accumulation threshold; it may instead reflect S100B’s short circulating half-life and the temporal resolution of sampling [27,34].

### 5.3. S100B: Critical Appraisal

S100B illustrates a specificity problem distinct from, and in some respects more severe than, those affecting the other analytes reviewed in this article. Because S100B is also expressed in peripheral tissues, including adipocytes, chondrocytes, and melanocytes, and because BBB permeability itself is modulated by factors unrelated to head trauma, serum S100B is confounded by extracranial sources and physiological states that independently affect barrier integrity or peripheral S100B production. Most notably for an athlete population, intense exercise can increase blood S100B concentrations in the absence of head impact, making non-contact exercise controls important in experimental design [33]. This exercise confound has been proposed, in a Letter to the Editor rather than a primary research report, as part of a broader hypothesis for why sports-related injury contexts may differ biologically from other injury mechanisms, although that hypothesis remains unresolved [33]. More fundamentally for this review, S100B’s evidence base in chronic CTE remains thin. The autoantibody-DTI-cognition pathway is mechanistically compelling but has not been tied directly to postmortem CTE pathology, and the available seasonal studies show acute responses without demonstrating a persistent cumulative signal [27,32,34]. S100B is therefore best regarded as a well-studied acute BBB-disruption and subconcussive-impact-associated marker whose relevance to chronic CTE diagnosis remains inferential.

## 6. Soluble TREM2 (sTREM2)

### 6.1. sTREM2: Rationale and Biological Mechanism

Triggering receptor expressed on myeloid cells 2 (TREM2) is an innate immune receptor expressed on the surface of microglia and macrophages that governs microglial activation, phagocytosis, and survival in response to tissue injury. Its soluble form, sTREM2, is generated by proteolytic shedding of the receptor’s extracellular domain or by alternative mRNA splicing and is detectable in both CSF and plasma. Unlike the other analytes reviewed in this article, sTREM2 does not index a structural injury product (tau, neurofilament, or an astrocytic/glial structural protein) but rather the activation state of microglia themselves, positioning it as a marker of the neuroinflammatory response to RHI rather than of the downstream structural damage that response may produce. sTREM2 carries an extensive validation history in Alzheimer’s disease, where it has been used to track microglial activation across disease stages and has shown associations with tau accumulation and amyloid pathology, but its application to CTE and RHI populations remains comparatively early-stage [18].

### 6.2. sTREM2: Evidence in RHI and CTE Cohorts

The foundational CTE-relevant study examined CSF concentrations of total tau (t-tau), phosphorylated tau, amyloid-β1-42, and sTREM2 in former NFL players reporting cognitive, behavioral, or mood changes, compared with unexposed controls. No significant between-group differences emerged for any of the four analytes, including sTREM2. Within the former-player group, however, a cumulative head impact index predicted higher CSF t-tau concentrations, and higher sTREM2 levels were positively associated with higher t-tau concentrations [18]. In simultaneous equation models the mediation ran in both directions: sTREM2 mediated the association between the exposure index and t-tau (*p* = 0.004), t-tau mediated the association between the exposure index and sTREM2 (*p* = 0.0196), and there was a statistically significant feedback loop between t-tau and sTREM2. The exposure index was modeled with a direct effect on t-tau only, so its relation to sTREM2 is indirect. The authors concluded that microglial activation may be both a cause and a consequence of neuronal injury after RHI. These cross-sectional data, from 68 former NFL players and 21 controls in the DETECT cohort, therefore do not support a one-directional sequence from exposure through microglial activation to tau; they position sTREM2 as a mechanistic correlate that is reciprocally related to tau rather than as a standalone diagnostic discriminator [18].

### 6.3. sTREM2: Critical Appraisal

sTREM2 offers a mechanistically distinct contribution to the fluid biomarker landscape reviewed in this article: where p-tau indexes tauopathy directly, NfL indexes axonal injury, GFAP indexes astrocytic injury, and S100B indexes BBB disruption, sTREM2 captures an aspect of innate immune and microglial activation. This mechanistic distinctiveness is a genuine strength for panel-based approaches, discussed further in Section 8.2. However, the evidence base specific to CTE and RHI remains limited to a modestly sized foundational study with a null primary case–control result; the positive sTREM2-t-tau finding is a secondary within-group association rather than a replicated case–control discrimination result [18]. Furthermore, sTREM2 assays and reference ranges have been developed primarily in Alzheimer’s disease cohorts, and whether an elevated value in an RHI-exposed individual reflects RHI-related microglial activation rather than age-related or comorbid AD-related neuroinflammation has not been rigorously disentangled. sTREM2 is therefore best regarded as a promising but preliminary mechanistic correlate whose current value lies primarily in clarifying pathophysiological relationships rather than in standalone diagnostic discrimination.

## 7. Comparative Synthesis

Table 1 summarizes the five analytes reviewed above across the sample matrix, the strength and character of their CTE/RHI-specific evidence base, the best available discrimination performance, and each marker’s principal limitation. No single analyte reviewed here satisfies the combination of CTE-specificity, replicated diagnostic accuracy, and mechanistic clarity that would be required of a standalone clinical test; each offers a partial, mechanistically distinct window onto the RHI-to-CTE pathophysiological cascade, reinforcing the rationale, developed further in Section 8.2, for multi-marker panel approaches rather than continued single-analyte optimization.

Before comparing analytes, the provenance of this evidence should be stated. References [9,13,14,15,24,28] all describe or report analyses of the DIAGNOSE CTE cohort of approximately 230–240 participants, and ref. [29] partially overlaps it. Patterns that appear to converge across several analyses therefore largely reflect one cohort examined from different angles rather than independent replication. A second cluster is likewise non-independent: the extracellular-vesicle studies [16,17], the sTREM2 study [18] and the plasma t-tau and exosomal tau studies [11,12] all derive from the DETECT cohort of former NFL players. Most of the evidence reviewed here therefore rests on two American football cohorts. Evidence independent of both is limited to the professional combat-athlete cohort [26], the single-season German football cohort [27], the college and high-school S100B studies [32,34] and the former rugby player cohort [19,20]. The footnote to Table 1 lists the source cohort of each supporting study. This non-independence should be kept in mind throughout the synthesis that follows.

Several patterns emerge from this synthesis. First, there is a broad inverse relationship between an analyte’s maturity as a general neurological biomarker and its demonstrated specificity to CTE: p-tau and S100B have long validation histories in AD and acute TBI, respectively, but neither provides a convincing CTE-specific signal once major confounds are considered. Second, biomarker timing is a major design challenge, but, as set out in Section 5.2, the available evidence does not establish a defined accumulation threshold for NfL, GFAP, or S100B. S100B, for example, can rise acutely after individual games while showing no persistent pre- to post-season elevation [34]. Short study windows may therefore be poorly matched to CTE’s multi-decade latency for some biological processes, while other analytes may simply have kinetics that favor acute rather than cumulative detection. Third, sTREM2 illustrates a distinct pattern: its current evidence is primarily correlational and mechanistic, linking microglial activation with t-tau within exposed participants rather than providing case–control discrimination [18]. Fourth, no analyte reviewed here has been validated against neuropathologically confirmed CTE in a sample large enough to generate stable sensitivity and specificity estimates. Most DIAGNOSE CTE findings are benchmarked against TES clinical diagnosis, CTE certainty categories, or Aβ-PET status rather than autopsy-confirmed CTE stage, because neuropathological correlation is available only for the small subset of participants who have died.

Interpreting the discrimination statistics reported above requires some context. By widely used conventions, an AUC of 0.70–0.80 indicates acceptable discrimination, 0.80–0.90 excellent discrimination, and 0.90 or above outstanding discrimination [35]. These labels describe group-level separation rather than clinical utility. An AUC of 0.72–0.74, as reported for p-tau181, p-tau231 and EV-derived tau [15,16,17], means that a randomly selected exposed individual has roughly a 72–74% probability of a higher value than a randomly selected control; at any single cutoff this implies substantial distributional overlap and a high false-positive rate, particularly where the target condition has low prevalence in the tested population. Such markers are inadequate as standalone diagnostics but may still contribute within a panel, provided they index biology not already captured by other components. The higher values reported in the literature, such as an AUC of 0.88 for p-tau217 or 0.91 for a multidomain model, were achieved against amyloid-PET status or TES classification rather than confirmed CTE [13,29], and none has been externally validated for CTE diagnosis.

### 7.1. Relationship of Fluid Biomarkers to CTE Neuropathology

**Figure 1:**
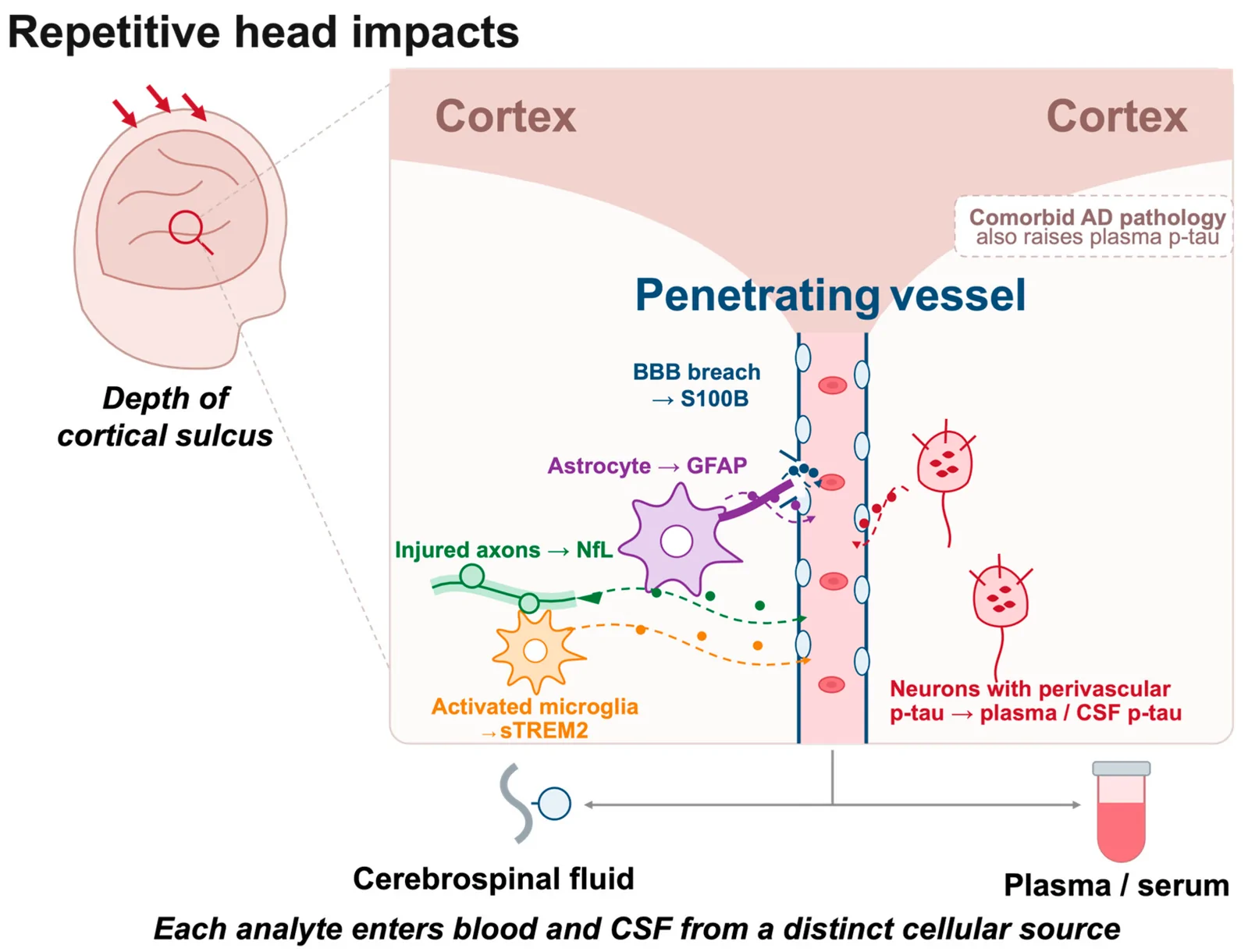
Cellular sources of fluid biomarkers at the depth of the cortical sulcus. Repetitive head impacts produce injury concentrated at the sulcal depths, where the pathognomonic CTE lesion develops around penetrating vessels. Blood–brain barrier disruption releases S100B; reactive astrogliosis releases GFAP; activated microglia shed sTREM2; injured axons release NfL; and neurons bearing perivascular hyperphosphorylated tau are the presumed source of fluid p-tau species. Each analyte enters blood and cerebrospinal fluid from a distinct cellular source, and plasma p-tau is additionally raised by comorbid Alzheimer’s disease pathology. Cell morphologies and molecular release are schematic and are not drawn to scale. Red arrows indicate repetitive head impacts; color-coded dashed arrows trace each analyte from its cellular source into the vessel and onward to cerebrospinal fluid and plasma/serum.

The pathognomonic CTE lesion is perivascular p-tau in neurons, with or without astrocytes, at the depths of the cortical sulci [4]. Across stages I–IV, tau pathology spreads from focal frontal lesions to widespread cortical, limbic and brainstem involvement, accompanied by axonal loss, astrogliosis and microglial activation [2]. The analytes reviewed here map onto these features unevenly (Table 2, Figure 1). Only plasma p-tau217 has been compared directly with autopsy-confirmed CTE stage, and it did not track stage [13]. For the remaining analytes, links to specific lesions are inferred from biological plausibility or extrapolated from AD and acute TBI data rather than established through CTE autopsy correlation, and the expected relations to disease stage summarized in Table 2 should therefore be read as hypotheses for future testing.

### 7.2. Mixed Pathology as a Limit on Interpretability

CTE rarely occurs in isolation, and this places a ceiling on what any of the analytes reviewed here can establish. In the foundational staging series, CTE was the sole diagnosis in 63% of cases, with comorbid motor neuron disease in 12%, Alzheimer’s disease in 11%, Lewy body disease in 16% and frontotemporal lobar degeneration in 6%; TDP-43 immunoreactive inclusions and neurites were present in 85% of cases, ranging from focal pathology in stages I–III to widespread involvement in stage IV [2]. Subsequent work in a larger brain-bank series found TDP-43 inclusions in 43.3% of CTE cases and hippocampal sclerosis in 23.4% and showed through structural equation modeling that RHI exposure years were associated with hippocampal TDP-43 inclusions through increased CTE stage [36]. Repetitive trauma therefore appears to promote co-accumulation of tau, TDP-43 and α-synuclein rather than tau alone.

The consequences for fluid biomarkers are direct. NfL and GFAP are proteinopathy-blind: they index axonal degeneration and astroglial activation irrespective of which protein drives them. Fluid p-tau assays read only the tau compartment. None of the five analytes can therefore distinguish CTE from CTE with comorbid TDP-43, α-synuclein or AD pathology, and a positive result in an RHI-exposed individual is compatible with any combination of these. Mixed pathology also offers a plausible alternative explanation for several findings discussed above. The NfL associations restricted to participants aged 60 and over (Section 3.2) and the exposure-linked GFAP findings concentrated in older participants (Section 4.2) are both consistent with accumulating age-related co-pathology rather than with an RHI-specific signal, and the available data cannot separate these interpretations. Resolving this would require panels that include markers of non-tau proteinopathies alongside the analytes reviewed here; no CTE fluid biomarker study has yet attempted this.

## 8. Challenges and Future Directions

### 8.1. Assay Standardization and Cross-Platform Harmonization

Cross-study comparability is limited by heterogeneous assay platforms, antibodies, sample-handling procedures, and cutoffs. These methods remain insufficiently harmonized, in contrast to the more advanced standardization efforts for Aβ42/40 and p-tau assays in the AD field. Several analytes reviewed here, notably p-tau217, borrow AD assay infrastructure, which accelerates translation but leaves CTE-relevant cutoffs largely unvalidated in RHI-exposed populations. Harmonized preanalytical protocols, reference materials, and cross-laboratory proficiency testing are prerequisites for the multisite validation recommended by the Leon Thal Summit [37].

### 8.2. Multi-Marker Panels over Single-Analyte Optimization

The synthesis in Section 7 argues against reliance on single-analyte approaches: each biomarker captures a distinct facet of RHI pathophysiology, and none alone has demonstrated validated clinical utility for CTE. As in AD, where a multi-protein CSF panel outperformed individual established biomarkers across multiple cohorts [38], early extracellular-vesicle proteomic work in RHI populations [16,17] supports combinatorial strategies. More recent evidence strengthens this rationale: a 2026 multidomain analysis combining cognition, plasma NfL, GFAP, total tau, p-tau231, APOE-ε4 status, and structural MRI achieved an AUC of 0.91 in the Professional Athletes Brain Health Study and 0.84 in DIAGNOSE CTE for TES classification [29]. These results support integrated frameworks while also emphasizing an important limitation: the outcome was TES, not neuropathologically confirmed CTE. Future panels should therefore be optimized and validated against autopsy-confirmed disease rather than clinical syndrome alone.

These figures require a caveat beyond the choice of outcome. The multidomain model includes cognitive performance as a predictor, while cognitive impairment is itself one of the core clinical features required for a TES diagnosis [5]. The reported discrimination is therefore subject to incorporation bias and should not be read as the performance of a biomarker panel. A biomarker-only model, or one in which the cognitive component is withheld, would be required to estimate the incremental contribution of the fluid analytes; such an estimate is not reported in the source study. The same caution applies to any future panel benchmarked against a clinical syndrome whose criteria overlap the predictors.

### 8.3. Longitudinal, Antemortem-to-Autopsy Cohort Infrastructure

The greatest structural limitation here is the near-absence of prospective cohorts linking antemortem biomarker trajectories to autopsy-confirmed CTE stage. DIAGNOSE CTE remains the field’s largest such investment, but autopsy correlation covers only a small subset of participants, most of whom are still living. The Leon Thal Summit flagged this tissue scarcity as rate-limiting and called for expanded brain-donation infrastructure and standardized exposure/outcome measurement across cohorts such as the Professional Athletes Brain Health Study to enable pooled analysis [37].

### 8.4. Toward CTE-Selective Assay Development

As detailed in Section 2.4, no analyte reviewed has an assay engineered for the CTE-specific molecular signature, its distinctive perivascular, sulcal-depth p-tau conformation or characteristic glial activity patterns, rather than generic disease markers. Characterizing CTE-specific tau conformers, rather than continuing to adapt AD-validated p-tau epitopes, may be necessary to close the specificity gap affecting every p-tau finding in this review.

### 8.5. Demographic and External-Validity Limitations

The cited evidence remains predominantly male and U.S.-based, with major contributions from former American football players and professional combat athletes. Generalizability to women, other contact-sport populations such as soccer and rugby, military blast exposure, and more racially and ethnically diverse cohorts remains limited. Current biomarker cutoffs and effect estimates should therefore not be assumed to transport directly across populations. Expanding research beyond the historically football-dominated evidence base remains a substantive requirement for external validity, even as newer fighter and rugby cohorts have begun to broaden the field [19,20,26,29].

Biological sex is a particularly important gap. CTE was first confirmed neuropathologically in a female professional athlete only in 2023, in a former Australian rules footballer with low-stage disease [39]. Available evidence suggests that biomarker responses to head impacts may themselves differ by sex. In a prospective study of Australian rules footballers, serum NfL, GFAP and tau rose after concussion in male but not female players, although the female subgroup was small and the authors called for adequately powered studies in women [40]. Experimental work indicates that axons from females are structurally smaller and contain fewer microtubules, rendering them more vulnerable to traumatic injury, which offers a candidate mechanism for sex-dependent release of axonal biomarkers [41]. Sex hormones, menstrual-cycle phase, body composition and differences in exposure history could each affect baseline concentrations, injury responses, or both. As participation by women in contact sports continues to grow, sex-stratified reference ranges and adequately powered female cohorts will be required before any fluid biomarker can be applied across sexes.

### 8.6. A Hypothetical Temporal Model of Biomarker Evolution

By analogy with the hypothetical model of dynamic biomarkers proposed for AD [42], the evidence reviewed here is compatible with, but does not establish, a three-phase working hypothesis. In an acute phase, S100B rises transiently after individual exposures [34]. In a cumulative-exposure phase, such transient responses recur over years of play, potentially accompanied by S100B autoantibody formation [32]. In a chronic neurodegenerative phase, elevations of NfL and GFAP would accompany axonal loss and astrogliosis [15,26], although GFAP was not raised in former rugby players [19,20], and any signal would be increasingly confounded by age and AD co-pathology [13,24]. No GFAP or sTREM2 time course has been measured in an RHI cohort; the suggestion that sTREM2 is highest during active exposure and declines once exposure ends is speculation offered in the Discussion of [18], not data. The model is hypothesis-generating only, and it can be tested solely in cohorts with serial sampling from active play to autopsy (Section 8.3).

## 9. Conclusions

Nearly two decades after CTE entered mainstream neurological and public consciousness, the disease still cannot be definitively diagnosed during life. This review has synthesized current evidence for five fluid biomarkers proposed to help close that gap—p-tau, NfL, GFAP, S100B, and sTREM2. The resulting picture is neither a diagnostic breakthrough nor a story of uniform failure, but a set of mechanistically distinct analytes that capture different components of the biological response to repetitive head impacts. None is individually sufficient, and none has yet been validated at the scale or against the neuropathological ground truth required for clinical translation.

Several conclusions follow directly from the comparative synthesis in Section 7. First, diagnostic specificity to CTE, as opposed to Alzheimer’s disease, general neurodegeneration, or acute traumatic injury, remains the field’s central unresolved challenge, and no analyte reviewed here has demonstrated both disease specificity and replicated diagnostic accuracy sufficient for clinical use. Second, chronicity and exposure timing are important methodological considerations: markers such as S100B can show clear acute sensitivity to individual head-impact exposure without producing a persistent season-long signal [34]. Frequent null findings in short-duration studies may therefore partly reflect mismatches between sampling design, biomarker kinetics, and CTE’s long latency, but, as set out in Section 5.2, current evidence does not establish a defined biomarker accumulation threshold. Third, no biomarker reviewed here has been validated against a large, prospectively followed, autopsy-confirmed sample, and this remains a major infrastructural gap explicitly recognized by the 2025 Leon Thal Summit consensus panel [37].

The trajectory implied by this evidence base points away from continued single-analyte optimization and toward integration. Combinatorial panels that pair NfL and GFAP with complementary inflammatory, tau-related, imaging, and genetic information may outperform individual fluid analytes, a direction explored in a recent multidomain TES analysis across two athlete cohorts, although its reported discrimination is subject to the incorporation bias discussed in Section 8.2 [29]. However, improved TES classification should not be conflated with diagnosis of neuropathologically confirmed CTE. Realizing the clinical potential of integrated models will require harmonized assay platforms and reference standards, expanded and coordinated brain-donation programs to grow autopsy-confirmed validation samples, and large, long-duration, multi-population studies.

The stakes of closing this gap extend well beyond diagnostic elegance. Without an antemortem biomarker, clinicians cannot offer athletes, military personnel, and their families individualized, biologically grounded risk information; therapeutic trials cannot enroll biologically confirmed cases or use biomarker trajectory as an outcome measure; and the substantial diagnostic overlap between CTE and other late-life neurodegenerative conditions cannot be resolved for the individual patient sitting in a clinic, only inferred at the population level from autopsy series.

## Figures and Tables

**Table 1:** Comparative summary of fluid biomarkers for antemortem CTE detection.

Biomarker	Sample Matrix	Primary Mechanism	Strength of CTE/RHI Evidence	Best Reported Discrimination	Principal Limitation	Refs	Reference Standard
p-tau (181/217/231)	Plasma, CSF	Hyperphosphorylated tau reflecting tauopathy burden	DIAGNOSE CTE: p-tau217 not linked to TES/CTE certainty or RHI metrics; signal strongly tracks Aβ pathology; DETECT: plasma t-tau linked to RHI index, exosomal tau higher in players; rugby: p-tau217 higher and linked to TES	AUC 0.742 for p-tau181/231 [15]; p-tau217 AUC 0.88 for Aβ-PET, not CTE	AD co-pathology confound; no validated fluid assay selective for CTE-specific tau	[11,12,13,14,15,16,17,19,20]	Unexposed controls; TES/CTE certainty; Aβ-PET; autopsy (*n* = 9)
NfL	Plasma, CSF	Axonal injury/neurodegeneration, nonspecific	DIAGNOSE CTE: linked to NBD symptoms and executive dysfunction, mainly in older participants	No validated CTE case–control AUC	Nonspecific; strong age confound; weak relation to quantitative RHI metrics	[15,24]	Unexposed controls; TES symptom measures
GFAP	Plasma, serum	Astrocytic injury/reactive astrogliosis	Football: higher in possible/probable vs. no/suggestive TES-CTE; fighters: higher GFAP linked to TES; season-long null study; rugby: not raised, lower on multiplex assay	No standalone CTE AUC; included in integrated TES models	Nonspecific astroglial marker; TES is not autopsy CTE; chronic kinetics unresolved	[15,19,20,26,27,28,29]	TES/CTE certainty; no autopsy data
S100B	Serum	Astrocytic protein indexing blood–brain barrier disruption	Acute post-game rises linked to impact frequency/kinematics; no cumulative season-long elevation	Validated for acute TBI/BBB use; no chronic CTE AUC	Exercise/extracranial confounds; short half-life; limited chronic CTE evidence	[27,30,31,32,33,34]	Pre- vs. post-impact sampling; no autopsy data
sTREM2	CSF, plasma	Microglial activation/innate immune response	No case–control difference; RHI index predicted t-tau, with bidirectional mediation between sTREM2 and t-tau	No case–control AUC; correlational only	Limited evidence; AD-derived assays; mechanistic correlate, not standalone diagnostic	[18]	Unexposed controls; within-group correlation

Source cohorts: DIAGNOSE CTE [13,14,15,24,28,29]; DETECT [11,12,16,17,18]; Professional Athletes Brain Health Study [26,29]; Advanced Brain Health Clinic rugby cohort [19,20]; other independent cohorts [27,32,34]. Refs. [30,31,33] are reviews or commentary.

**Table 2:** Hypothesized relationship of fluid biomarkers to CTE neuropathological features. Entries in the second and third columns are untested hypotheses based on biological plausibility; only the fourth column reports data.

Biomarker	Neuropathological Feature Most Plausibly Indexed	Hypothesized Relation to CTE Stage (Untested)	Direct Autopsy Correlation in CTE
p-tau (181/217/231)	Neuronal and perivascular p-tau; comorbid AD tau	Hypothesized rise with stage; not observed for p-tau217	Yes (*n* = 9); p-tau217 did not track stage [13]
NfL	Axonal degeneration, white matter rarefaction	Hypothesized rise in advanced stages	None
GFAP	Reactive astrogliosis; thorn-shaped astrocytes	Unknown	None
S100B	Blood–brain barrier disruption, astroglial perturbation (acute)	Unlikely to track chronic stage	None
sTREM2	Microglial activation, neuroinflammation	Unknown; no time-course data	None

## Data Availability

No new data were created or analyzed in this study. Data sharing is not applicable to this article. All data discussed are drawn from previously published sources, which are cited throughout the text and listed in the References section.

## Abbreviations

The following abbreviations are used in this manuscript:

CTE	Chronic traumatic encephalopathy
RHIs	Repetitive head impacts
TES	Traumatic encephalopathy syndrome
AD	Alzheimer’s disease
p-tau	Phosphorylated tau
NfL	Neurofilament light chain
GFAP	Glial fibrillary acidic protein
S100B	S100 calcium-binding protein B
sTREM2	Soluble triggering receptor expressed on myeloid cells 2
CSF	Cerebrospinal fluid
BBB	Blood–brain barrier
TBI	Traumatic brain injury
AUC	Area under the receiver operating characteristic curve
NBD	Neurobehavioral dysregulation
t-tau	Total tau
DETECT	Diagnosing and Evaluating Traumatic Encephalopathy using Clinical Tests
EV	Extracellular vesicle

## References

[1] McKee A.C., Cantu R.C., Nowinski C.J., Hedley-Whyte E.T., Gavett B.E., Budson A.E., Santini V.E., Lee H.S., Kubilus C.A., Stern R.A. (2009). Chronic Traumatic Encephalopathy in Athletes: Progressive Tauopathy after Repetitive Head Injury. J. Neuropathol. Exp. Neurol..

[2] McKee A.C., Stein T.D., Nowinski C.J., Stern R.A., Daneshvar D.H., Alvarez V.E., Lee H.S., Hall G., Wojtowicz S.M., Baugh C.M. (2013). The Spectrum of Disease in Chronic Traumatic Encephalopathy. Brain.

[3] Mez J., Daneshvar D.H., Kiernan P.T., Abdolmohammadi B., Alvarez V.E., Huber B.R., Alosco M.L., Solomon T.M., Nowinski C.J., McHale L. (2017). Clinicopathological Evaluation of Chronic Traumatic Encephalopathy in Players of American Football. JAMA.

[4] McKee A.C., Stein T.D., Huber B.R., Crary J.F., Bieniek K., Dickson D., Alvarez V.E., Cherry J.D., Farrell K., Butler M. (2023). Chronic Traumatic Encephalopathy (CTE): Criteria for Neuropathological Diagnosis and Relationship to Repetitive Head Impacts. Acta Neuropathol..

[5] Katz D.I., Bernick C., Dodick D.W., Mez J., Mariani M.L., Adler C.H., Alosco M.L., Balcer L.J., Banks S.J., Barr W.B. (2021). National Institute of Neurological Disorders and Stroke Consensus Diagnostic Criteria for Traumatic Encephalopathy Syndrome. Neurology.

[6] Mez J., Dwyer B., Alosco M.L., Yew B., Pritchett A., Bernal Fernández N., Hicks A.J., Abdolmohammadi B., Durape S., Uretsky M. (2025). Validity of the 2021 Traumatic Encephalopathy Syndrome Criteria for CTE Pathology. Alzheimer’s Dement..

[7] Qi B., Tan J., Feng D., Guan L., Li J., Cao M., Zou Y. (2025). Prevalence of Chronic Traumatic Encephalopathy in Athletes With Repetitive Head Impacts: A Systematic Review and Meta-Analysis. Scand. J. Med. Sci. Sports.

[8] Butler M., Pervaiz N., Breen K., Calderazzo S., Ypsilantis P., Wang Y., Breda J.C., Mazzilli S., Nicks R., Spurlock E. (2025). Repeated Head Trauma Causes Neuron Loss and Inflammation in Young Athletes. Nature.

[9] Alosco M.L., Mariani M.L., Adler C.H., Balcer L.J., Bernick C., Au R., Banks S.J., Barr W.B., Bouix S., Cantu R.C. (2021). Developing Methods to Detect and Diagnose Chronic Traumatic Encephalopathy During Life: Rationale, Design, and Methodology for the DIAGNOSE CTE Research Project. Alzheimer’s Res. Ther..

[10] Alosco M.L., Culhane J., Mez J. (2021). Neuroimaging Biomarkers of Chronic Traumatic Encephalopathy: Targets for the Academic Memory Disorders Clinic. Neurotherapeutics.

[11] Alosco M.L., Tripodis Y., Jarnagin J., Baugh C.M., Martin B., Chaisson C.E., Estochen N., Song L., Cantu R.C., Jeromin A. (2017). Repetitive Head Impact Exposure and Later-Life Plasma Total Tau in Former National Football League Players. Alzheimer’s Dement. Diagn. Assess. Dis. Monit..

[12] Stern R.A., Tripodis Y., Baugh C.M., Fritts N.G., Martin B.M., Chaisson C., Cantu R.C., Joyce J.A., Shah S., Ikezu T. (2016). Preliminary Study of Plasma Exosomal Tau as a Potential Biomarker for Chronic Traumatic Encephalopathy. J. Alzheimer’s Dis..

[13] Miner A.E., Zetterberg H., Blennow K., Groh J.R., Singh A., Dieckhoff K., Tripodis Y., Adler C.H., Balcer L.J., Bernick C. (2026). Plasma Phosphorylated Tau 217 in Participants at Risk for Chronic Traumatic Encephalopathy. JAMA Netw. Open.

[14] Miner A.E., Ashton N.J., Zetterberg H., Blennow K., Groh J.R., Tripodis Y., Adler C., Balcer L., Bernick C.B., Peskind E.R. (2025). The Utility of Plasma P-Tau217 in Former American Football Players at Risk for Chronic Traumatic Encephalopathy. Alzheimer’s Dement..

[15] Miner A.E., Groh J.R., Tripodis Y., Adler C.H., Balcer L.J., Bernick C., Zetterberg H., Blennow K., Peskind E., Ashton N.J. (2024). Examination of Plasma Biomarkers of Amyloid, Tau, Neurodegeneration, and Neuroinflammation in Former Elite American Football Players. Alzheimer’s Dement. J. Alzheimer’s Assoc..

[16] Muraoka S., DeLeo A.M., Yang Z., Tatebe H., Yukawa-Takamatsu K., Ikezu S., Tokuda T., Issadore D., Stern R.A., Ikezu T. (2021). Proteomic Profiling of Extracellular Vesicles Separated From Plasma of Former National Football League Players at Risk for Chronic Traumatic Encephalopathy. Aging Dis..

[17] Muraoka S., Jedrychowski M.P., Tatebe H., DeLeo A.M., Ikezu S., Tokuda T., Gygi S.P., Stern R.A., Ikezu T. (2019). Proteomic Profiling of Extracellular Vesicles Isolated From Cerebrospinal Fluid of Former National Football League Players at Risk for Chronic Traumatic Encephalopathy. Front. Neurosci..

[18] Alosco M.L., Tripodis Y., Fritts N.G., Heslegrave A., Baugh C.M., Conneely S., Mariani M., Martin B.M., Frank S., Mez J. (2018). Cerebrospinal Fluid Tau, Aβ, and sTREM2 in Former National Football League Players: Modeling the Relationship Between Repetitive Head Impacts, Microglial Activation, and Neurodegeneration. Alzheimer’s Dement. J. Alzheimer’s Assoc..

[19] Graham N.S.N., Zimmerman K.A., Hain J., Rooney E., Lee Y., Del Giovane M., Parker T., Wilson M.G., Malhotra P., David M.C.B. (2025). Biomarker Evidence of Neurodegeneration in Mid-Life Former Rugby Players. Brain.

[20] Graham N., Zimmerman K., Hain J., Rooney E., Lee Y., Del Giovane M., Parker T., Wilson M., Patel M., Veleva E. (2026). Midlife Plasma Proteomic Profiles Indicate Altered Amyloid and Tau Processing in Former Elite Rugby Players. J. Neurol. Neurosurg. Psychiatry.

[21] Falcon B., Zivanov J., Zhang W., Murzin A.G., Garringer H.J., Vidal R., Crowther R.A., Newell K.L., Ghetti B., Goedert M. (2019). Novel Tau Filament Fold in Chronic Traumatic Encephalopathy Encloses Hydrophobic Molecules. Nature.

[22] Kraus A., Saijo E., Metrick M.A., Newell K., Sigurdson C.J., Zanusso G., Ghetti B., Caughey B. (2019). Seeding Selectivity and Ultrasensitive Detection of Tau Aggregate Conformers of Alzheimer Disease. Acta Neuropathol..

[23] Metrick M.A., Ferreira N.d.C., Saijo E., Kraus A., Newell K., Zanusso G., Vendruscolo M., Ghetti B., Caughey B. (2020). A Single Ultrasensitive Assay for Detection and Discrimination of Tau Aggregates of Alzheimer and Pick Diseases. Acta Neuropathol. Commun..

[24] van Amerongen S., Pulukuri S.V., Tuz-Zahra F., Tripodis Y., Cherry J.D., Bernick C., Geda Y.E., Wethe J.V., Katz D.I., Alosco M.L. (2024). Inflammatory Biomarkers for Neurobehavioral Dysregulation in Former American Football Players: Findings From the DIAGNOSE CTE Research Project. J. Neuroinflamm..

[25] U.S. Food and Drug Administration (2018). Decision Memorandum: Banyan Brain Trauma Indicator (DEN170045). https://www.accessdata.fda.gov/cdrh_docs/reviews/DEN170045.pdf.

[26] Conway Kleven B.D., Chien L.-C., Cross C.L., Labus B., Bernick C. (2025). Traumatic Encephalopathy Syndrome: Head Impact Exposure and Blood Biomarkers in Professional Combat Athletes. J. Head Trauma Rehabil..

[27] Bastgen T., Evers J., Oedekoven C., Weide C., Herzog L., Ashton N., Zetterberg H., Blennow K., Albus A., Vidovic N. (2024). Repetitive Head Injuries in German American Football Players Do Not Change Blood-Based Biomarker Candidates for CTE During a Single Season. Neurol. Res. Pract..

[28] Miner A.E., Groh J.R., Tripodis Y., Adler C., Balcer L., Bernick C.B., Zetterberg H., Blennow K., Ashton N.J., Peskind E.R. (2024). CSF Biomarkers and Supportive Psychiatric Features of the TES Criteria in Former American Football Players. Alzheimer’s Dement..

[29] Conway Kleven B.D., Chien L.-C., Surwill D., Alosco M.L., Wethe J.V., Tripodis Y., Adler C.H., Shenton M.E., Pasternak O., Katz D.I. (2026). Cognitive, Biomarker, and Neuroimaging Indices Associated with Traumatic Encephalopathy Syndrome Across Two Independent Athlete Cohorts. Alzheimer’s Res. Ther..

[30] Oris C., Kahouadji S., Durif J., Bouvier D., Sapin V. (2023). S100B, Actor and Biomarker of Mild Traumatic Brain Injury. Int. J. Mol. Sci..

[31] Tharmaratnam T., Iskandar M.A., Tabobondung T.C., Tobbia I., Gopee-Ramanan P., Tabobondung T.A. (2018). Chronic Traumatic Encephalopathy in Professional American Football Players: Where Are We Now?. Front. Neurol..

[32] Marchi N., Bazarian J.J., Puvenna V., Janigro M., Ghosh C., Zhong J., Zhu T., Blackman E., Stewart D., Ellis J. (2013). Consequences of Repeated Blood-Brain Barrier Disruption in Football Players. PLoS ONE.

[33] Butler T. (2023). Letter to the Editor: Intense Exercise and Associated Blood-Brain Barrier Leakiness and Systemic Inflammation at the Time of Brain Injury May Be Relevant to Understanding Why Only Sports-Related Concussions Are Associated With Chronic Traumatic Encephalopathy. J. Neurotrauma.

[34] Zonner S.W., Ejima K., Bevilacqua Z.W., Huibregtse M.E., Charleston C., Fulgar C., Kawata K. (2019). Association of Increased Serum S100B Levels With High School Football Subconcussive Head Impacts. Front. Neurol..

[35] Hosmer D.W., Lemeshow S. (2000). Applied Logistic Regression.

[36] Nicks R., Clement N.F., Alvarez V.E., Tripodis Y., Baucom Z.H., Huber B.R., Mez J., Alosco M.L., Aytan N., Cherry J.D. (2023). Repetitive Head Impacts and Chronic Traumatic Encephalopathy Are Associated with TDP-43 Inclusions and Hippocampal Sclerosis. Acta Neuropathol..

[37] Bernick C., Kleven B.C., Alosco M.L., Arciniega H., Ashton N., Bender A., Bieniek K.F., Cordes D., Cordes L., Katz D.I. (2026). Advancing Biomarker Development for Chronic Traumatic Encephalopathy: Summary and Recommendations From the 2025 Leon Thal Summit. Alzheimer’s Dement. J. Alzheimer’s Assoc..

[38] Hou X., Qiu Y., Li H., Yan Y., Zhao D., Ji S., Ni J., Zhang J., Liu K., Qing H. (2025). Machine-Learning Based Strategy Identifies a Robust Protein Biomarker Panel for Alzheimer’s Disease in Cerebrospinal Fluid. Alzheimer’s Res. Ther..

[39] Suter C.M., Affleck A.J., Pearce A.J., Junckerstorff R., Lee M., Buckland M.E. (2023). Chronic Traumatic Encephalopathy in a Female Ex-Professional Australian Rules Footballer. Acta Neuropathol..

[40] McDonald S.J., O’Brien W.T., Symons G.F., Chen Z., Bain J., Major B.P., Costello D., Yamakawa G., Sun M., Brady R.D. (2021). Prolonged Elevation of Serum Neurofilament Light after Concussion in Male Australian Football Players. Biomark. Res..

[41] Dollé J.-P., Jaye A., Anderson S.A., Ahmadzadeh H., Shenoy V.B., Smith D.H. (2018). Newfound Sex Differences in Axonal Structure Underlie Differential Outcomes from In Vitro Traumatic Axonal Injury. Exp. Neurol..

[42] Jack C.R., Knopman D.S., Jagust W.J., Shaw L.M., Aisen P.S., Weiner M.W., Petersen R.C., Trojanowski J.Q. (2010). Hypothetical Model of Dynamic Biomarkers of the Alzheimer’s Pathological Cascade. Lancet Neurol..

